# Performance Analysis of Dual-Hop AF Relaying with Non-Linear/Linear Energy Harvesting

**DOI:** 10.3390/s22165987

**Published:** 2022-08-10

**Authors:** Mohammadreza Babaei, Lütfiye Durak-Ata, Ümit Aygölü

**Affiliations:** 1 Informatics Institute, Istanbul Technical University, 34469 Istanbul, Turkey; 2 Department of Electronics and Communication Engineering, Istanbul Technical University, 34469 Istanbul, Turkey

**Keywords:** AF relaying, BEP analysis, dual-hop, energy harvesting, IoT

## Abstract

Massive device-to-device communication nodes and Internet of Things (IoT) devices are expected to be crucial components in next-generation wireless networks. However, the energy constraint of these nodes presents a challenge since the energy of the batteries is limited. Motivated by this, radio frequency energy harvesting (EH) has been developed as an efficient strategy to overcome the energy constraint of IoT devices and sensor networks. In this paper, a wireless-powered dual-hop amplify-and-forward relaying system, in the absence of a direct link between the source (S) and the destination (D), is considered. It is assumed that a dedicated power beacon (PB) transmits an energy-bearing signal from which the power-constrained S and relay (R) harvest energy. Theoretical derivations of bit error probability, outage probability, and throughput expressions are performed for both linear and non-linear energy harvesting models. Moreover, the theoretical results provided for different system parameters are validated via Monte Carlo simulations. The obtained results reveal the difference between the realistic non-linear EH model and the conventional linear EH model, which overestimates the system performance at high levels of harvested energy. Thus, it leads to misunderstanding the real performance of the EH systems. However, at low levels of harvested energy, both models behave similarly and provide realistic results.

## 1. Introduction

By deploying massive sensor and Internet of Things (IoT) devices in next-generation wireless communication systems, energy limitation is revealed as one of the challenges for IoT devices. Normally, IoT devices are empowered by a battery, which limits their capacity and constrains their operational time. However, radio frequency (RF) EH can be an effective solution for empowering IoT devices, leading to increased operational time for battery-free IoT devices. In the literature, simultaneous wireless information and power transfer (SWIPT) and wireless-powered communication (WPC) schemes are considered as RF energy harvesting methods for the power-constrained nodes [1,2,3,4,5,6,7]. Power-splitting (PS) and time-switching (TS) are two EH receiver structures utilized in the SWIPT scheme. In PS EH mode, the power-constraint node harvests power from the incoming signal energy where one portion of the signal power is used for harvesting energy while the remainder is used for information processing. Moreover, in TS EH mode, two non-overlapping time intervals are dedicated to EH and IP, respectively. Moreover, the amount of harvested energy is considered as a linear or non-linear function of the energy receiving (ER) node input power [2,4,8,9,10,11,12,13,14].

### Related Works

The system performance of power-constrained nodes considering the linear EH model is investigated in [15,16,17,18,19,20,21,22], where the harvested energy is a linear function of the received power at the battery-less node. However, due to the non-linearity of the EH circuit in practice, non-linear EH models are investigated in [23,24,25,26,27,28,29,30,31,32,33,34,35,36,37,38,39,40,41,42,43,44].

A massive multiple-input multiple-output (MIMO) relaying system with PS EH mode is investigated in an IoT cooperative network in [15]. The considered system’s achievable sum-rate is obtained for a relay which is power-constrained and harvests its power using the linear EH model. The system performance of power-constrained smart devices is investigated in [16], where a TS EH mode with the linear EH model is employed. A full-duplex (FD) dual-hop (DH) relaying network with the linear EH model is studied in [17]. Here, the power-constraint relay node applies PS and TS modes for EH purposes. Moreover, the relay forwards the source node signal to the destination, applying both amplify-and-forward (AF) and decode-and-forward (DF) relaying. Comprehensive throughput analyses of the considered system are performed for both the PS and TS EH modes. The throughput of the considered system is maximized for both PS and TS EH modes. The system performance of a cognitive IoT network with AF/DF relaying is investigated in [18], where the IoT network accesses the spectrum using orthogonal frequency-division multiplexing (OFDM). Moreover, the linear EH model with PS EH mode is considered in [18]. A cooperative non-orthogonal multiple access (NOMA) overlay spectrum sharing system with power-constrained secondary transmitters (STs) is examined in [19], where the STs operate in FD mode. Moreover, the outage probability and system throughput are investigated considering the linear EH model and PS EH mode. In [20], the outage probability of a cooperative DH relaying system with the linear EH model is derived where the TS EH mode is investigated. Here, the communication between the source and NOMA IoT devices is provided through power-constrained relay nodes.

Maximization of harvested energy in a non-convex problem is investigated in [23]. Here, an unmanned aerial vehicle (UAV) transfers power to the ground user location where the non-linear EH model is considered. The outage probability and throughput of a DH AF relaying two-way (TW) system are investigated in [24], where the relay harvests energy from both source nodes through the TS mode for the non-linear EH model. The throughput and ergodic capacity for a non-linear, piece-wise model are considered in [25], where a power-constrained source node harvests its power from the destination. The performance of a DH AF relaying system with a non-linear EH model is investigated in [26]. A closed-form expression for the bit error probability (BEP) is derived for binary differential phase-shift keying modulation, where the relay is assumed to be power-constrained. The performance of a cooperative AF relaying system is analyzed in [27]. The outage probability is investigated for perfect and imperfect channel state information (CSI), where the relay applies PS for the non-linear EH model. A massive MIMO SWIPT system’s energy efficiency is maximized in [28] where a base station transmits power, and the TS EH mode ratio is optimized. The spectral efficiency and average harvested energy of an FD DF relaying network with multiple users are investigated in [29].

The performance of an FD cognitive radio (CR) system for both linear and non-linear EH models is analyzed in [30]. It is assumed that the secondary transmitter is power-constrained and harvests its energy from both the primary transmitter (PT) and the secondary receiver. The bit error rate (BER) performance for both primary and secondary users is derived for different system parameters. The performance of a multi-antenna FD CR EH system is investigated in [31], where the transmitter employs the NOMA technique to transmit from the PT. Both the throughput and the outage probability of the system based on the non-linear EH model are investigated. In [32], the system performance of a cooperative NOMA network, which consists of three nodes, is analyzed. The near user is considered to be power-constrained and harvests its power from the incoming source signal. The outage probability of an overlay CR is considered in [33]. It is assumed that the secondary user is power-constrained and harvests its energy from the PT signal. The system performance of a DH DF relaying system with multiple power-constrained relays is investigated in [34]. A closed-form outage probability expression is derived considering the TS EH protocol. A cooperative DF relaying network with spectrum sensing and an ER node is examined in [35]. The power-constrained node simultaneously harvests energy and processes information by applying the PS technique.

A MIMO wireless power transfer ER architecture design is studied in [36]. The total harvested power is maximized for the proposed generic architecture of multiple ER nodes. The rate-energy trade-off of a point-to-point SWIPT system with a non-linear EH model is investigated in [37]. The ergodic fading channel is considered in [37] where the power-constrained node applies the PS EH mode. The sum throughput of the relay-based wireless-powered system is investigated in [38]. The relay adopts the non-linear EH model to harvest power from a dedicated power beacon. The sum throughput is maximized jointly considering a non-convex problem with parameters of power and time fraction. A non-linear EH model for a device-to-device network is proposed in [39]. In this study, the energy efficiency is maximized, considering the PS EH mode of the IoT nodes by optimizing the resource and power allocation. A WPC network with a non-linear EH model is investigated in [40], where the weighted sum of computation bits in each user is maximized for the considered non-convex optimization problem. A cooperative DH AF relaying system with a mixed fading environment and a non-linear EH model is investigated in [41]. Here, it is assumed that the relay is power-constrained and harvests its power from the source using PS/TS EH modes. The system performance of a MIMO IoT network in the presence of cooperative jamming is considered in [42], where a non-linear EH model with PS protocol is assumed. In [43], the non-linear EH model is applied to UAV-assisted FD IoT networks, where infinite and finite blocklength codes are considered. The performance of the system is analyzed in terms of the block error rate, where the theoretical derivation is performed considering Rician shadowed fading channels. Our study can be extended for finite blocklength considering [43] and references therein.

## 2. Methods

In this paper, the theoretical expressions for the bit error probability (BEP) and the throughput of a wireless-powered dual-hop amplify-and-forward (WP DH AF) relaying system are considered. Moreover, we investigate a non-linear piece-wise EH model which is mathematically tractable in terms of the PDF, CDF of harvested power, and the system performance. Apart from the linear EH model described in the literature, this model provides practical performance analyses. Specifically, the non-linear EH model considered in [8,11,26,27,34,45] is addressed since this model is mathematically tractable compared to other non-linear EH models [9,10,12,13]. In contrast to the linear EH model, which overestimates system performance, the considered non-linear piece-wise EH model provides realistic system performance along with the practical non-linear EH models. Since most papers in the literature have considered the linear EH model, in this paper, the effect of the linear EH model is investigated along with the non-linear piece-wise EH model, to provide a comprehensive system analysis. Hence, comparisons of linear and non-linear EH models are also provided. To the best of the authors’ knowledge, the BEP, outage probability, and throughput of the considered system have not yet been investigated in the literature. The contributions of this paper are summarized as follows:

- Theoretical derivation of BEP expressions considering both linear and non-linear EH models is performed.
- Throughputs of the considered system for both linear and non-linear EH models are derived.
- Simulation and theoretical results of the considered system are provided for different system parameters, such as distance, power, and achievable rate.

The paper is organized as follows: The system model is described in Section 3. Theoretical BEP derivations are provided in Section 4. Section 5 deals with the throughput analysis. Numerical results are presented in Section 6. Finally, Section 7 concludes the paper.

## 3. System Model

**Figure 1:**
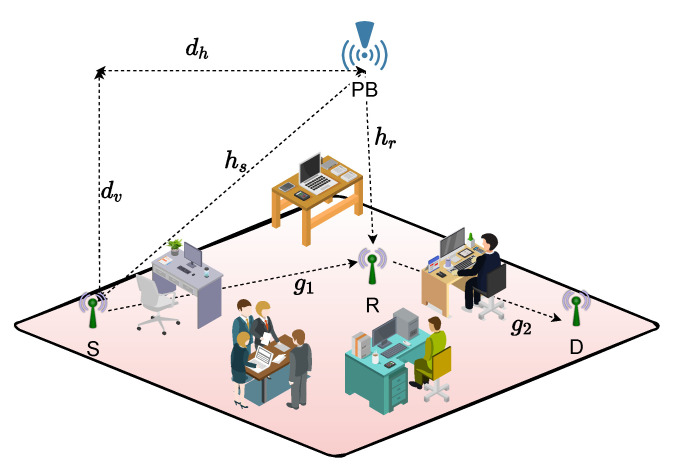
Wireless-powered DH AF relaying system model where S and R harvest their energy from a dedicated PB. S and R use their harvested power for data transmission.

The DH AF relaying system under consideration is given in Figure 1, where the source (S) and relay (R) are power-constrained nodes and harvest their energy from a dedicated power beacon (PB). Hence, S and R use their harvested powers for data transmission. In the absence of a direct link between S and the destination (D), the communication is provided with the help of R. All nodes are equipped with only one antenna.

**Figure 2:**
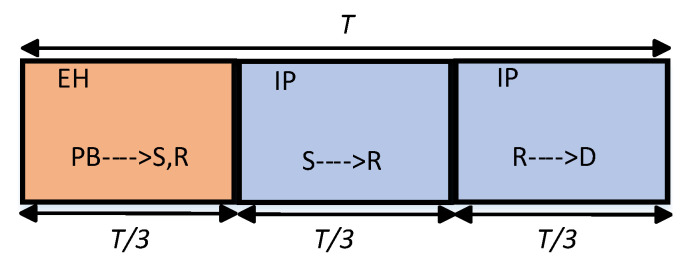
Transmission time frame of the considered wireless-powered system. The transmission frame is assumed to be equally-partitioned into three time slots for EH and information processing (IP).

A typical RF EH process is achieved in a short range and a line of sight (LoS) exists between PB and the EH nodes. Inspired by this fact, we assume that the links between nodes in Figure 1 are exposed to mix-fading [30,46]. The proper fading channel model, including an LoS component, is considered as the Rician model. However, the cumulative distribution function (CDF) and probability distribution function (PDF) of the Rician distribution include special functions which make the analysis more complicated and the results are not mathematically tractable. On the other hand, the Nakagami-*m* fading model provides a good approximation of the Rician channel model [47,48,49]. Motivated by this, we assume that the links PB→S and PB→R are exposed to Nakagami-*m* fading, represented by channel coefficients $h_{s}$ and $h_{r}$, with channel parameters $m_{z}$ and $m_{w}$, respectively. Moreover, the links S→R and R→D are subject to Rayleigh fading, with gains $g_{1}$ and $g_{2}$, respectively. It is assumed that the CSI is perfectly known at the receiving nodes, and the channels are assumed to be exposed to flat fading and remain fixed during a transmission interval and vary independently from one interval to another. The overall transmission time *T* is divided into three equal time slots, as shown in Figure 2. It should be noted that this assumption is considered in [2,4] where it provides a maximized system performance. In the first time slot of duration T/3, PB broadcasts the dedicated energy bearing signal. In the second and third time slots, S and R transmit their signals to R and D, respectively. $n_{r}$ and $n_{d}$ denote additive white Gaussian noise (AWGN) samples which are independent and identically distributed (i.i.d.) complex Gaussian random variables (r.v) as $n_{r},n_{d}∼CN\left(0,N_{0}\right)$. Finally, the notation and system parameters are listed in Table 1 and Table 2, respectively.

### Linear and Non-Linear EH Models

In the literature, two EH models are considered: linear and non-linear. In the linear EH model, the transmit power of the power-constrained node is increased by increasing the harvested power at the considered node [2,4]. This causes a misrepresentation of the amount of harvested power [53] since, in practical EH circuits, the amount of the harvested power increases to a threshold level rather than the considered amount of harvested power in the linear EH model.

In other words, in practice, due to the non-linear behavior of the diodes in EH circuits, non-linear EH models are more realistic compared to the linear EH model. Additionally, a maximum threshold power is defined for the non-linear EH model, such that, for harvested power greater than this threshold value, the transmit powers of S and R take this fixed threshold value [8,9,10,11,12,13]. Specifically, we assume the non-linear EH model given in [8,11] since the non-linear EH models proposed in [9,10,12,13] are not mathematically tractable. Moreover, the considered model provides a good approximation of practical EH circuits at low and high amounts of harvested power [8,53] which broadens the insight of EH system design along with the well-studied linear EH model.

In the first time slot, S and R harvest energy from PB. For the linear EH model, the harvested power for S and R is given as [2]

(1) $$\begin{array}{c} P_{i}=P_{h_{i}}=\eta_{i}P_{T}L_{it}{\left|h_{i}\right|}^{2}, \end{array}$$

where $P_{h_{i}}$ is the harvested power at node *i* with i∈{s,r}. However, assuming the non-linear EH model, the harvested power at S and R is given as [8,11]

(2) $$P_{i}=\left\{\begin{array}{cc} P_{h_{i}}, & P_{h_{i}}\leq P_{th_{i}}\\ P_{th_{i}}, & P_{h_{i}}>P_{th_{i}}, \end{array}\right.$$

where i∈{s,r}. We assume that all of the harvested energy at both nodes S and R during the first time slot of T/3 is consumed for the transmission of the signal *x* in the consecutive time slots of each T/3 since there is no available energy buffer at the power-constrained nodes. Moreover, high input power is limited to the threshold power $P_{th_{i}}$. In thesecond time slot, the received signal at node R is

(3) $$\begin{array}{c} y_{r}=\sqrt{P_{s}L_{sr}}g_{1}x+n_{r}. \end{array}$$

In the third time slot, R forwards the amplified version of the received signal in (3). The received signal at D is then given as

(4) $$\begin{array}{c} y_{d}=\sqrt{\frac{P_{r}L_{rd}}{G}}g_{2}y_{r}+n_{d}, \end{array}$$

where $G=E\{|y_{r}{|}^{2}\}=P_{s}L_{sr}{\left|g_{1}\right|}^{2}+N_{0}$ is the normalization factor. Substituting (3) in (4), we have

(5) $$\begin{array}{c} y_{d}=\sqrt{\frac{P_{s}P_{r}L_{sr}L_{rd}}{G}}g_{1}g_{2}x+\sqrt{\frac{P_{r}L_{rd}}{G}}g_{2}n_{r}+n_{d}. \end{array}$$

The received SNR at D is calculated from (5) as

(6) $$\begin{array}{c} \gamma =\frac{XY}{X+Y+1}\leq \min\left(X,Y\right), \end{array}$$

where $X=ZX^{\prime }$, $Y=WY^{\prime }$ and we assume a tight upper bound in (6). For simplicity, we assume $Z=P_{s}$, $W=P_{r}$, $X^{\prime }=L_{sr}{\left|g_{1}\right|}^{2}/N_{0}$ and $Y^{\prime }=L_{rd}{\left|g_{2}\right|}^{2}/N_{0}$. In addition, $Z∼\Gamma (m_{z},\phi_{z})$ and $W∼\Gamma (m_{w},\phi_{w})$ are Gamma distributed r.v.s with parameters $m_{z}$ and $m_{w}$, respectively, and the scaling parameters are $\phi_{z}=m_{z}/\bar{z}$ and $\phi_{w}=m_{w}/\bar{w}$. Moreover, $\bar{z}=\eta_{s}P_{T}L_{st}\Omega_{h_{s}}$ and $\bar{w}=\eta_{r}P_{T}L_{rt}\Omega_{h_{r}}$. Furthermore, $X^{\prime }$ and $Y^{\prime }$ are exponentially distributed as $\mathrm{Exp}({\bar{x}}^{\prime })$ and $\mathrm{Exp}({\bar{y}}^{\prime })$, where ${\bar{x}}^{\prime }=L_{sr}\Omega_{g_{1}}/N_{0}$ and ${\bar{y}}^{\prime }=L_{rd}\Omega_{g_{2}}/N_{0}$, respectively.

In (6), using ([54], eq. 6-81), the conditioned CDF of γ is expressed as

(7) $$\begin{array}{c} F_{\gamma }\left(\gamma |Z,W\right)=F_{X}\left(\gamma |Z\right)+F_{Y}\left(\gamma |W\right)-F_{X}\left(\gamma |Z\right)F_{Y}\left(\gamma |W\right), \end{array}$$

where

(8) $$\begin{array}{cc} \; & F_{X}\left(\gamma |Z\right)=1-\exp\left(-\frac{\gamma }{Z{\bar{x}}^{\prime }}\right), \end{array}$$

(9) $$\begin{array}{cc} \; & F_{Y}\left(\gamma |W\right)=1-\exp\left(-\frac{\gamma }{W{\bar{y}}^{\prime }}\right). \end{array}$$

Substituting both (8) and (9) in (7) and simplifying, the conditioned CDF in (7) is obtained as

(10) $$\begin{array}{c} F_{\gamma }\left(\gamma |Z,W\right)=1-\exp\left(-\gamma \left(\frac{1}{Z{\bar{x}}^{\prime }}+\frac{1}{W{\bar{y}}^{\prime }}\right)\right). \end{array}$$

## 4. BEP Analysis

In this section, the BEP of the considered DH AF relaying system is derived for both non-linear and linear EH models. In order to obtain the BEP expression, the analytical expression of symbol error probability (SEP) is first derived. The overall BEP expression is given for high SNR, assuming the common approximation for Gray mapping [55] as $P_{b}^{i}≊P_{s}^{i}/\vartheta$. Here, i∈{L,NL}, L and NL represent linear and non-linear EH models, respectively. The conditioned SEP for both linear and non-linear EH models is calculated as [4]

(11) $$\begin{array}{c} P_{s}^{L,NL}\left(e|Z,W\right)=\frac{a\sqrt{b}}{2\sqrt{\pi }}\int_{0}^{\infty }\frac{e^{-b\gamma }}{\sqrt{\gamma }}F_{\gamma }\left(\gamma |Z,W\right)d\gamma =\frac{a\sqrt{b}}{2\sqrt{\pi }}\left(I_{1}-I_{2}\left(Z,W\right)\right), \end{array}$$

where parameters *a* and *b* denote the modulation coefficients for *M*-PSK/QAM [4] and $F_{\gamma }\left(\gamma |Z,W\right)$ is given in (10). In (11),

(12) $$\begin{array}{c} I_{1}=\int_{0}^{\infty }\frac{e^{-b\gamma }}{\sqrt{\gamma }}d\gamma =\sqrt{\frac{\pi }{b}} \end{array}$$

and

(13) $$\begin{array}{c} I_{2}\left(Z,W\right)=\int_{0}^{\infty }\frac{1}{\sqrt{\gamma }}\exp\left(-\gamma \left(b+\frac{1}{Z{\bar{x}}^{\prime }}+\frac{1}{W{\bar{y}}^{\prime }}\right)\right)d\gamma =\sqrt{\frac{\pi }{b+\frac{1}{Z{\bar{x}}^{\prime }}+\frac{1}{W{\bar{y}}^{\prime }}}} \end{array}$$

which are calculated using ([50], eq. 3.361-2). After substituting (12) and (13) and simplifying, (11) is obtained as

(14) $$\begin{array}{cc} P_{s}^{L,NL}\left(e|Z,W\right) & =\frac{a}{2}\left[1-\frac{1}{\sqrt{1+\frac{1}{Z\bar{x}}+\frac{1}{W\bar{y}}}}\right], \end{array}$$

where $\bar{x}=b{\bar{x}}^{\prime }$ and $\bar{y}=b{\bar{y}}^{\prime }$.

### 4.1. BEP Analysis of the Non-Linear EH Model

In this subsection, the BEP of the non-linear EH model is derived analytically. The overall BEP for the non-linear EH model is expressed as

(15) $$\begin{array}{c} P_{b}^{NL}≊\left(P_{s1}+P_{s2}+P_{s3}+P_{s4}\right)/\vartheta , \end{array}$$

where $P_{s1}$, $P_{s2}$, $P_{s3}$, and $P_{s4}$ stand for the four states of power harvesting processes considering both nodes S and R, which are calculated as

(16) $$\begin{array}{cc} \; & P_{s1}=\int_{w=0}^{P_{th}}\underset{A(w)}{\underset{︸}{\int_{z=0}^{P_{th}}P_{s}^{L,NL}\left(e|z,w\right)f_{Z}\left(z\right)dz}}f_{W}\left(w\right)dw, \end{array}$$

(17) $$\begin{array}{cc} \; & P_{s2}=P_{s}^{L,NL}\left(e|\begin{array}{c} z=P_{th}\\ w=P_{th} \end{array}\right)\underset{B_{1}}{\underset{︸}{\int_{w=P_{th}}^{\infty }f_{W}\left(w\right)dw}}\underset{B_{2}}{\underset{︸}{\int_{z=P_{th}}^{\infty }f_{Z}\left(z\right)dz}}, \end{array}$$

(18) $$\begin{array}{cc} \; & P_{s3}=\underset{A_{1}{=A\left(w\right)|}_{w=P_{th}}}{\underset{︸}{\int_{z=0}^{P_{th}}P_{s}^{L,NL}\left(e|z,w\right)f_{Z}\left(z\right)dz}}\underset{B_{1}}{\underset{︸}{\int_{w=P_{th}}^{\infty }f_{W}\left(w\right)dw}}, \end{array}$$

and

(19) $$\begin{array}{cc} \; & P_{s4}=\underset{A_{2}{=A\left(z\right)|}_{z=P_{th}}}{\underset{︸}{\int_{w=0}^{P_{th}}P_{s}^{L,NL}\left(e|z,w\right)f_{W}\left(w\right)dw}}\underset{B_{2}}{\underset{︸}{\int_{z=P_{th}}^{\infty }f_{Z}\left(z\right)dz}}. \end{array}$$

A(w), and $P_{s1}$ in (16) and A(z) in (19) are calculated in Appendix A, Appendix B, and Appendix C, respectively.

In (19), $A_{2}$ is calculated from (A29) as $A_{2}{=A\left(z\right)|}_{z=P_{th}}$. Furthermore, using ([50], eq. 3.351-2), $B_{2}$ in (19) is calculated as

(20) $$\begin{array}{c} B_{2}=\int_{P_{th}}^{\infty }f_{Z}\left(z\right)dz=D\Gamma \left(m_{z},P_{th}\phi_{z}\right)/\phi_{z}^{m_{z}}, \end{array}$$

where

(21) $$\begin{array}{c} f_{Z}\left(z\right)=Dz^{m_{z}-1}\exp\left(-z\phi_{z}\right) \end{array}$$

with $D=\phi_{z}^{m_{z}}/\Gamma \left(m_{z}\right)$. Finally, $P_{s4}$ in (19) is calculated by substituting $A_{2}{=A\left(z\right)|}_{z=P_{th}}$ in (A29) and (20).

In (19), $A_{1}$ is calculated from (A2) as $A_{1}{=A\left(w\right)|}_{w=P_{th}}$. Moreover, using ([50], 3.351-2), $B_{1}$ in (18) is calculated as

(22) $$\begin{array}{c} B_{1}=\int_{P_{th}}^{\infty }f_{W}\left(w\right)dw=I\Gamma \left(m_{w},P_{th}\phi_{w}\right)/\phi_{w}^{m_{w}}, \end{array}$$

where

(23) $$\begin{array}{c} f_{W}\left(w\right)=Iw^{m_{w}-1}\exp\left(-w\phi_{w}\right) \end{array}$$

with $I=\phi_{w}^{m_{w}}/\Gamma \left(m_{w}\right)$. Moreover, $P_{s3}$ in (18) is calculated by substituting $A_{1}{=A\left(w\right)|}_{w=P_{th}}$ in (A2) and (22). Finally, $P_{s2}$ in (17) is calculated using (22), (20), and replacing $z=P_{th}$ and $w=P_{th}$ in (14).

### 4.2. Linear EH Model

In this subsection, the BEP of the considered DH AF relaying system is derived for the linear EH. Here, it is assumed that the harvested energy is linearly dependent on the received energy and increases by increasing the energy transferred from PB. At high SNR values, the overall BEP for the linear EH model is given as

(24) $$\begin{array}{c} P_{b}^{L}≊\frac{1}{\vartheta }\int_{0}^{\infty }\underset{P_{s}\left(e|w\right)}{\underset{︸}{\int_{0}^{\infty }P_{s}^{L,NL}\left(e|z,w\right)f_{Z}\left(z\right)dz}}f_{W}\left(w\right)dw. \end{array}$$

In (24), $P_{s}\left(e|w\right)$ is calculated by substituting $f_{Z}\left(z\right)$ and $P_{s}^{L,NL}\left(e|z,w\right)$ from (21) and (14) as

(25) $$\begin{array}{c} P_{s}\left(e|w\right)=\frac{a}{2}\left(E_{0}-F_{0}\left(w\right)\right), \end{array}$$

where

(26) $$\begin{array}{c} E_{0}=\int_{0}^{\infty }f_{Z}\left(z\right)dz=1, \end{array}$$

and after simplifying

(27) $$\begin{array}{c} F_{0}\left(w\right)=\frac{D}{\sqrt{B}}\int_{0}^{\infty }\frac{z^{m_{z}-0.5}}{\sqrt{1+z/G}}\exp\left(-z\phi_{z}\right)dz, \end{array}$$

where G=B/C. Using ([56], eq. 10), (27), is rewritten as

(28) $$\begin{array}{c} F_{0}\left(w\right)=\frac{D}{\Gamma \left(0.5\right)\sqrt{B}}\int_{0}^{\infty }z^{m_{z}-0.5}G\begin{array}{c} 11\\ 11 \end{array}\left(\frac{Z}{G}|\begin{array}{c} 0.5\\ 0 \end{array}\right)\exp\left(-z\phi_{z}\right)dz. \end{array}$$

Using ([50], eq. 7.813-1), and substituting G=B/C, (28) is obtained as

(29) $$\begin{array}{c} F_{0}\left(w\right)=\frac{D}{\Gamma \left(0.5\right)\sqrt{B}}\phi_{z}^{-(m_{z}+0.5)}G\begin{array}{c} 12\\ 21 \end{array}\left(\frac{1+w\bar{y}}{B\phi_{z}w\bar{y}}|\begin{array}{c} 0.5-m_{z},0.5\\ 0 \end{array}\right). \end{array}$$

Then, (25) is calculated by substituting (26) and (29). Moreover, substituting (25) and (23) in (24) we have

(30) $$\begin{array}{cc} P_{b}^{L} & ≊\frac{1}{\vartheta }\frac{a}{2}\int_{0}^{\infty }\left(1-F_{0}\left(w\right)\right)f_{W}\left(w\right)dw=\frac{a}{2\vartheta }\left(P-J\right), \end{array}$$

where

(31) $$\begin{array}{c} P=\int_{0}^{\infty }f_{W}\left(w\right)dw=1 \end{array}$$

and

(32) $$\begin{array}{c} J=\frac{DI}{\Gamma \left(0.5\right)\sqrt{B}}\phi_{z}^{-(m_{z}+0.5)}\int_{0}^{\infty }w^{m_{w}-1}\exp\left(-w\phi_{w}\right)G\begin{array}{c} 12\\ 21 \end{array}\left(\frac{1+w\bar{y}}{B\phi_{z}w\bar{y}}|\begin{array}{c} 0.5-m_{z},0.5\\ 0 \end{array}\right)dw. \end{array}$$

Please note that no closed-form solution is available for (32) as it is calculated numerically. Finally, the overall BEP for the linear EH model is obtained from (30) which depends on only *J* in (32).

## 5. Throughput Analysis

In this section, the throughput of the DH AF relaying system is calculated for the non-linear and linear EH models. The outage probability, defined as the probability that the target rate exceeds the instantaneous achievable rate, can be given as

(33) $$\begin{array}{c} P_{out}^{i}=P_{r}\left\{\frac{1}{3}\log_{2}\left(1+\gamma \right)<R\right\}=P_{r}\left\{\gamma <\gamma_{th}\right\}=F_{\gamma }\left(\gamma_{th}\right), \end{array}$$

where *R* is the target rate, $\gamma_{th}=2^{3R}-1$ is the threshold SNR, γ is the instantaneous SNR at D and $F_{\gamma }\left(\cdot \right)$ is the CDF of γ. The factor 1/3 is due to the transmission of one symbol per three time slots. Please note that i=NL and i=L stand for non-linear and linear EH models, respectively. The throughput of the considered system is calculated as

(34) $$\begin{array}{c} \tau^{i}=\frac{R}{3}\left(1-P_{out}^{i}\right). \end{array}$$

### 5.1. Outage Probability for the Non-Linear EH Model

The system outage probability for the non-linear EH model is calculated as

(35) $$\begin{array}{c} P_{out}^{NL}=P_{out_{1}}+P_{out_{2}}+P_{out_{3}}+P_{out_{4}}, \end{array}$$

where the four outage probabilities at the right-hand side are calculated as

(36) $$\begin{array}{cc} \; & P_{out_{1}}=\int_{w=0}^{P_{th}}\underset{Q(w)}{\underset{︸}{\int_{z=0}^{P_{th}}F_{\gamma }\left(\gamma |z,w\right)f_{Z}\left(z\right)dz}}f_{W}\left(w\right)dw, \end{array}$$

(37) $$\begin{array}{cc} \; & P_{out_{2}}=F_{\gamma }\left(\gamma |\begin{array}{c} z=P_{th}\\ w=P_{th} \end{array}\right)\underset{B_{1}}{\underset{︸}{\int_{w=P_{th}}^{\infty }f_{W}\left(w\right)dw}}\underset{B_{2}}{\underset{︸}{\int_{z=P_{th}}^{\infty }f_{Z}\left(z\right)dz}}, \end{array}$$

(38) $$\begin{array}{cc} \; & P_{out_{3}}=\underset{Q_{1}{=Q\left(w\right)|}_{w=P_{th}}}{\underset{︸}{\int_{z=0}^{P_{th}}F_{\gamma }\left(\gamma |z,w\right)f_{Z}\left(z\right)dz}}\underset{B_{1}}{\underset{︸}{\int_{w=P_{th}}^{\infty }f_{W}\left(w\right)dw}}, \end{array}$$

and

(39) $$\begin{array}{cc} \; & P_{out_{4}}=\underset{Q_{2}{=Q\left(z\right)|}_{z=P_{th}}}{\underset{︸}{\int_{w=0}^{P_{th}}F_{\gamma }\left(\gamma |z,w\right)f_{W}\left(w\right)dw}}\underset{B_{2}}{\underset{︸}{\int_{z=P_{th}}^{\infty }f_{Z}\left(z\right)dz}}. \end{array}$$

In (36), substituting $f_{Z}\left(z\right)$ and $f_{W}\left(w\right)$ from (21) and (23), respectively, and considering $F_{\gamma }\left(\gamma |z,w\right)$ in (10), we have

(40) $$\begin{array}{c} Q\left(w\right)=E_{1}-\exp\left(-\frac{\gamma }{w{\bar{y}}^{\prime }}\right)O_{1}\left(\gamma \right) \end{array}$$

and

(41) $$\begin{array}{c} P_{out_{1}}=E_{1}E_{2}-O_{1}\left(\gamma \right)O_{2}\left(\gamma \right). \end{array}$$

Here, $E_{1}$ and $E_{2}$ are calculated from (A3) and (A30), respectively, and

(42) $$\begin{array}{c} O_{1}\left(\gamma \right)=\int_{0}^{P_{th}}\exp\left(-\frac{\gamma }{z{\bar{x}}^{\prime }}\right)f_{Z}\left(z\right)dz \end{array}$$

and

(43) $$\begin{array}{c} O_{2}\left(\gamma \right)=\int_{0}^{P_{th}}\exp\left(-\frac{\gamma }{w{\bar{y}}^{\prime }}\right)f_{W}\left(w\right)dw. \end{array}$$

Substituting, $f_{Z}\left(z\right)$ and $f_{W}\left(w\right)$ from (21) and (23) in (42) and (43), respectively, and using ([50], eq. 1.211-1), ([56], eq. 11) and applying ([50], eq. 9.31-1) and ([50], eq. 9.31-2) for both (42) and (43), after simplification, using ([56], eq. 26), we have

(44) $$\begin{array}{c} O_{1}\left(\gamma \right)=D\sum_{t=0}^{\Upsilon }\frac{{\left(-\phi_{z}\right)}^{t}}{t!}P_{th}^{m_{z}+t}G\begin{array}{c} 12\\ 32 \end{array}\left(\frac{P_{th}{\bar{x}}^{\prime }}{\gamma }|\begin{array}{c} 1,1-(m_{z}+t),0\\ 0,-(m_{z}+t) \end{array}\right) \end{array}$$

and

(45) $$\begin{array}{c} O_{2}\left(\gamma \right)=I\sum_{t=0}^{\Upsilon }\frac{{\left(-\phi_{w}\right)}^{t}}{t!}P_{th}^{m_{w}+t}G\begin{array}{c} 12\\ 32 \end{array}\left(\frac{P_{th}{\bar{y}}^{\prime }}{\gamma }|\begin{array}{c} 1,1-(m_{w}+t),0\\ 0,-(m_{w}+t) \end{array}\right). \end{array}$$

In (44) and (45), Υ is a parameter determining the trade-off between complexity and accuracy. Finally, substituting (44) and (45) in (41), $P_{out_{1}}$ in (41) is derived. In order to calculate (37), we replace $z=w=P_{th}$ in $F_{\gamma }\left(\gamma |z,w\right)$ given in (10) and calculate $B_{1}$ and $B_{2}$ from (22) and (20), respectively. Moreover, for (38), $Q_{1}$ and $B_{1}$ are calculated by replacing $w=P_{th}$ in (40) and (22), respectively. Pursuing the same procedure for (40), (39) is expressed as

(46) $$\begin{array}{c} Q\left(z\right)=E_{2}-\exp\left(-\frac{\gamma }{z{\bar{x}}^{\prime }}\right)O_{2}\left(\gamma \right). \end{array}$$

Then, in (39), $Q_{2}$ and $B_{2}$ are calculated by replacing $z=P_{th}$ in (46) and (20), respectively. Finally, (35) is calculated by substituting (36), (37), (38) and (39). The throughput of the proposed system is derived by substituting (35) in (34).

### 5.2. Outage Probability for the Linear EH Model

The outage probability of the linear EH model is calculated as

(47) $$\begin{array}{c} P_{out}^{L}=\int_{0}^{\infty }F_{\gamma }\left(\gamma |W\right)f_{W}\left(w\right)dw, \end{array}$$

where

(48) $$\begin{array}{c} F_{\gamma }\left(\gamma |W\right)=\int_{0}^{\infty }F_{\gamma }\left(\gamma |Z,W\right)f_{Z}\left(z\right)dz \end{array}$$

and $F_{\gamma }\left(\gamma |Z,W\right)$ is given in (10). Substituting (21) and (10) and after simplification, (48) is obtained as

(49) $$\begin{array}{c} F_{\gamma }\left(\gamma |W\right)=S_{11}-S_{12}, \end{array}$$

where

(50) $$\begin{array}{c} S_{11}=\int_{0}^{\infty }f_{Z}\left(z\right)dz=1 \end{array}$$

and

(51) $$\begin{array}{cc} S_{12}= & D\exp\left(-\frac{\gamma }{w{\bar{y}}^{\prime }}\right)\int_{0}^{\infty }z^{m_{z}-1}\exp\left(-z\phi_{z}-\frac{\gamma }{z{\bar{x}}^{\prime }}\right)dz\\ = & 2D\exp\left(-\frac{\gamma }{w{\bar{y}}^{\prime }}\right){\left(\frac{\gamma }{{\bar{x}}^{\prime }\phi_{z}}\right)}^{0.5m_{z}}K_{m_{z}}\left(2\sqrt{\frac{\gamma \phi_{z}}{{\bar{x}}^{\prime }}}\right). \end{array}$$

Here, (51) is obtained by using ([50], eq. 3.471-9). Substituting (49) in (47) and simplifying, we have

(52) $$\begin{array}{c} P_{out}^{L}=S_{21}-S_{22}, \end{array}$$

where

(53) $$\begin{array}{c} S_{21}=S_{11}\int_{0}^{\infty }f_{W}\left(w\right)dw=S_{11}=1 \end{array}$$

and

(54) $$\begin{array}{c} S_{22}=2DI{\left(\frac{\gamma }{{\bar{x}}^{\prime }\phi_{z}}\right)}^{0.5m_{z}}K_{m_{z}}\left(2\sqrt{\frac{\gamma \phi_{z}}{{\bar{x}}^{\prime }}}\right)\int_{0}^{\infty }w^{m_{w}-1}\exp\left(-w\phi_{w}-\frac{\gamma }{w{\bar{y}}^{\prime }}\right)dw. \end{array}$$

Using ([50], eq. 3.471-9), after simplification, the CDF of the SNR at node D, namely, the outage probability is calculated as

(55) $$\begin{array}{cc} P_{out}^{L} & =1-4DI{\left(\frac{1}{{\bar{x}}^{\prime }\phi_{z}}\right)}^{0.5m_{z}}{\left(\frac{1}{{\bar{y}}^{\prime }\phi_{w}}\right)}^{0.5m_{w}}\gamma^{0.5(m_{z}+m_{w})}\\ & \times K_{m_{z}}\left(2\sqrt{\frac{\gamma \phi_{z}}{{\bar{x}}^{\prime }}}\right)K_{m_{w}}\left(2\sqrt{\frac{\gamma \phi_{w}}{{\bar{y}}^{\prime }}}\right). \end{array}$$

Finally, the throughput of the linear EH model is obtained by substituting (55) in (34).

## 6. Results and Discussion

In this section, the simulation and theoretical results are presented for different system parameters, which provides a comprehensive insight into the analysis of the WP DH AF relaying system. In all figures, the simulation and theoretical curves are denoted by symbols and lines, respectively. The numerical results obtained from simulations and theoretical derivations are in perfect match with each other at high SNR values, which verifies our theoretical analysis. Moreover, throughout the paper, the integrals in (32), and (A21) are numerically calculated using MATLAB and Wolfram Mathematica Computer Software. Furthermore, the numerical results for the derived summations in analytical expressions are obtained for Υ=200, which makes them sufficiently reliable. The BER results are provided with respect to various system parameters. The numerical results for BEP are obtained from (15) and (30) and for throughput by replacing (35) and (55) in (34), for the linear and non-linear EH models, respectively. Unless otherwise stated, we assume $d_{v}=1$ and $\eta_{s}=\eta_{r}=0.9$. The path loss coefficient is taken as ν=2.7 [57]. All channel gains and noise powers are fixed as Ω=1 and $N_{0}=1$, respectively. Moreover, R is located at the middle of S and D, so that $d_{sr}=d_{rd}=d_{sd}/2$. It is assumed that S, R, and D are located co-linearly, and PB moves along a trajectory parallel to S-R-D. Then, $d_{st}=\sqrt{d_{v}^{2}+d_{h}^{2}}$ and $d_{rt}=\sqrt{d_{v}^{2}+{\left(d_{sr}-d_{h}\right)}^{2}}$. Please note that the above assumptions are for numerical examples; the derived analytical expressions are obtained for the general case, and the nodes can be randomly located. The channel parameters for the links S→PB and R→PB are chosen as $m_{z}=2$ and $m_{w}=2$, respectively. For the BEP analysis, we provide the results for 4-QAM modulation, and for rates R=1,2,3 bit/sec/Hz, which are valid for low energy power-constraint nodes. Please note that the approximation in (6) provides a tight upper bound at medium and high SNR values for the following BER results.

**Figure 3:**
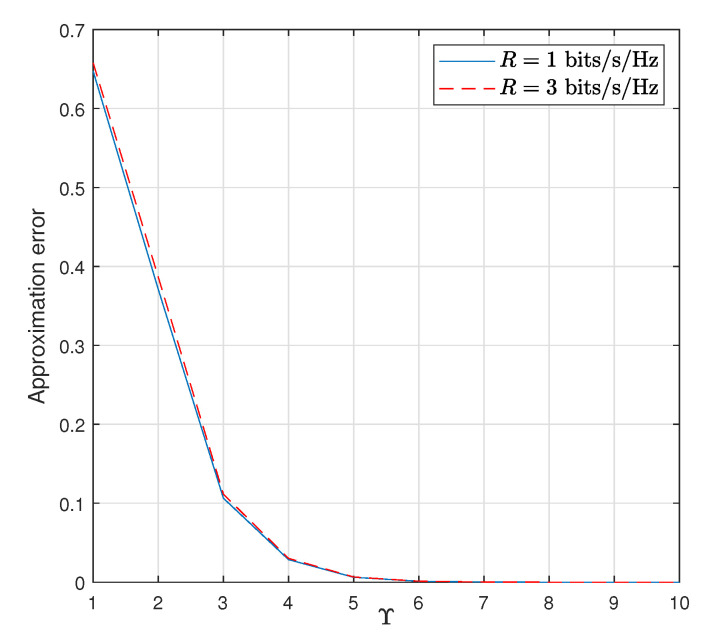
Relative approximation error versus parameter Υ of the WP DH AF relaying system for $P_{T}=40$ dB, and $P_{th}=35$ dB.

**Figure 4:**
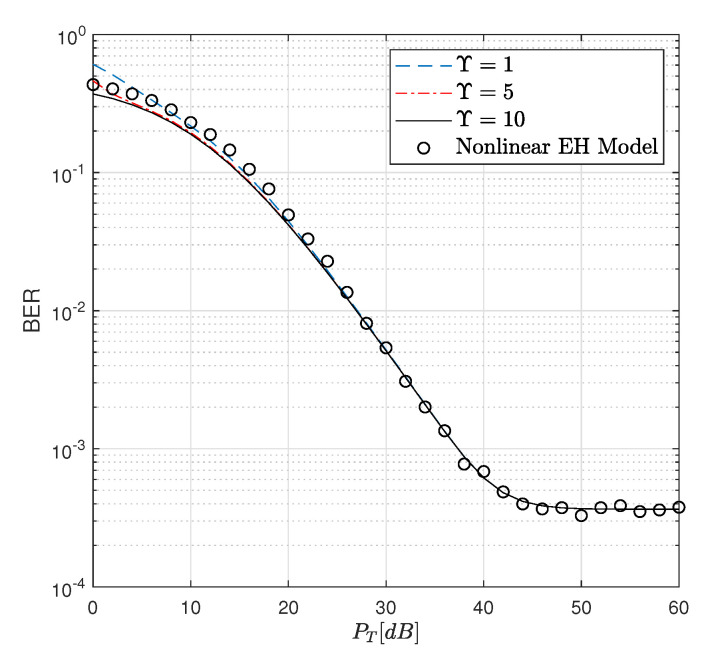
BER performance of the WP DH AF relaying system versus $P_{T}$ dB for $P_{th}=35$ dB.

Figure 3 represents the relative approximation error of the throughput with respect to the parameter Υ. Specifically, we define

(56) $$\begin{array}{c} \varepsilon =\left|\frac{\mathrm{analytical}\;\mathrm{value}-\mathrm{simulation}\;\mathrm{value}}{\mathrm{simulation}\;\mathrm{value}}\right| \end{array}$$

as the amount of relative error. Here the simulation value is obtained by the Monte Carlo method while the analytical value is calculated from (35). As seen from Figure 3, for both R=1 and R=3 bits/sec/Hz, by increasing the value of Υ, the error is decreased and tends to zero. Moreover, the results are sufficiently accurate when they are obtained for Υ=7, which provides an error value of ε=0.0002. However, we guaranteed the results by taking Υ=200. In addition, the BER results are depicted in Figure 4 for different values of Υ considering (15). From Figure 4, it is concluded that Υ=10 provides more accurate upper bound values compared to Υ=1.

**Figure 5:**
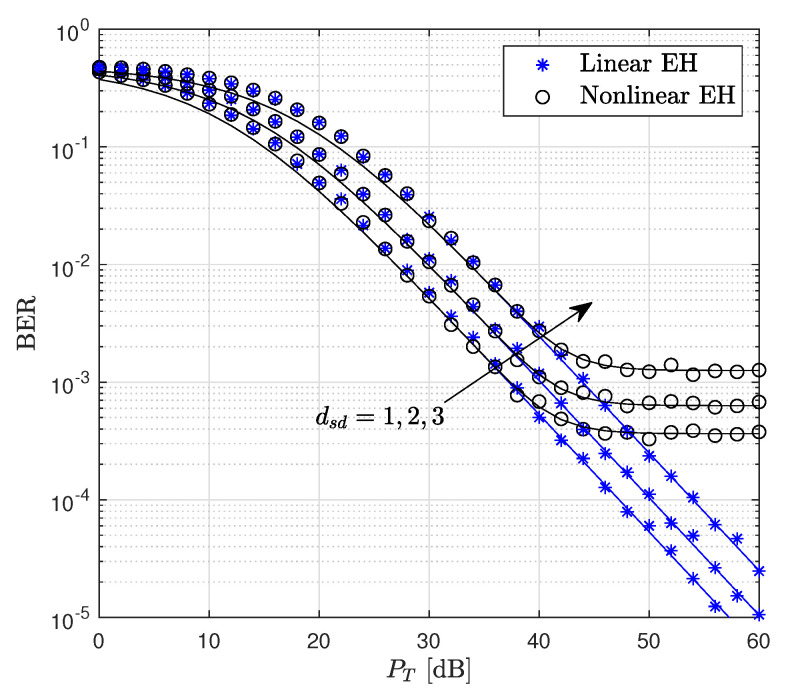
BER performance of the WP DH AF relaying system versus $P_{T}$ dB where $P_{th}=35$ dB.

The BER performance of the proposed WP DH AF relaying system with respect to $P_{T}$ is presented in Figure 5. For $P_{T}$ values lower than 40 dB, the linear and non-linear models provide the same performance, while for $P_{T}$ higher than 40 dB, the BER performance for the non-linear EH model converges to the error floor, which verifies that the harvested power is higher than the threshold power. In other words, for higher SNR values, $P_{s}=P_{r}=P_{th}$. It is seen from Figure 5 that the linear EH model overestimates the system performance compared to the realistic results obtained for the non-linear EH model. Moreover, considering the linear EH model, approximately, 2 dB, and 6 dB SNR losses are obtained for the BER of $10^{-3}$ by increasing the value of $d_{sd}$ from 1 to 2 and 3, respectively.

**Figure 6:**
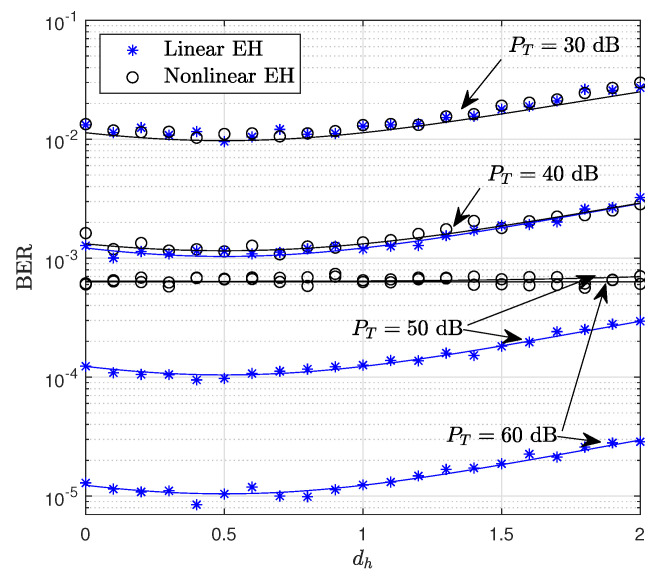
BER performance of the WP DH AF relaying system versus $d_{h}$ ($m_{w}=m_{z}=2$, $P_{T}=\left\{30,40,50,60\right\}$ dB).

Figure 6 shows the BER performance versus $d_{h}$, where it is assumed that $d_{sd}=2$. It is seen from Figure 6 that the results provided for $P_{T}=30$ dB and $P_{T}=40$ dB are similar for both linear and non-linear EH models, since the threshold power in the non-linear EH model is assumed as $P_{th}=35$ dB, and the amount of harvested energy in both models is equal. However, for higher values of $P_{T}$, the linear EH model outperforms the non-linear EH where the amount of harvested power is saturated at $P_{th}=35$ dB, which weakens the system performance compared to the linear EH model. Please note that for $P_{T}=50$ dB and $P_{T}=60$ dB, the results provided for the non-linear EH model are approximately the same, since for higher input power of PB, $P_{s}$ and $P_{r}$ are limited by $P_{th}=35$ dB.

**Figure 7:**
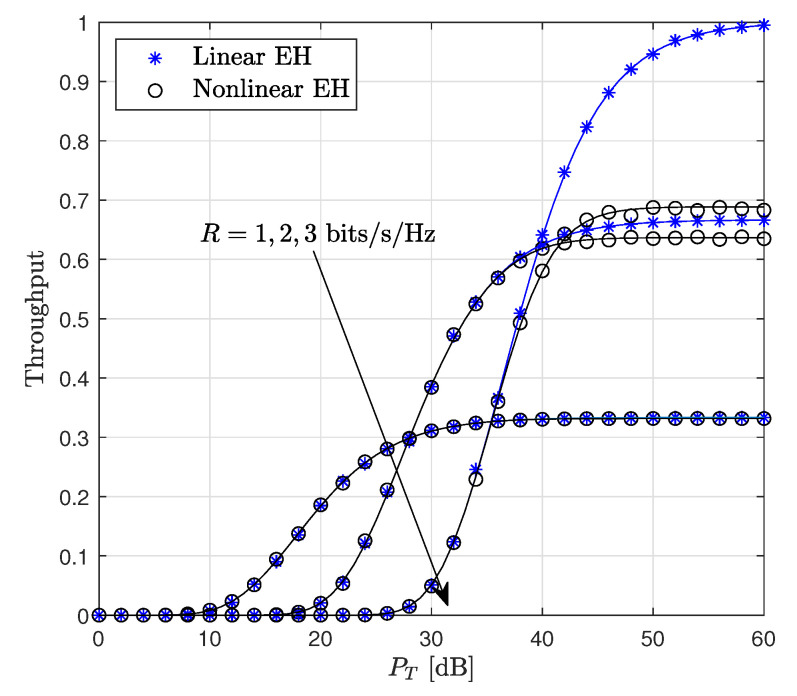
Throughput performance of the WP DH AF relaying system versus $P_{T}$ ($d_{sd}=1$, $P_{th}=35$ dB).

The throughput of the considered system versus $P_{T}$ for $d_{sd}=1$ is illustrated in Figure 7. It is shown that for a target rate value of R=1 bit/sec/Hz, the linear and non-linear EH models provide approximately the same performance, while for both R=2 and R=3, the linear EH model has higher throughput compared to the non-linear EH model. This is based on the fact that the linear EH model causes a misrepresentation of the system performance compared to the considered EH model since the amount of the harvested energy at both S and R nodes is miscalculated. Hence, this provides a misunderstanding of the design of the EH systems.

**Figure 8:**
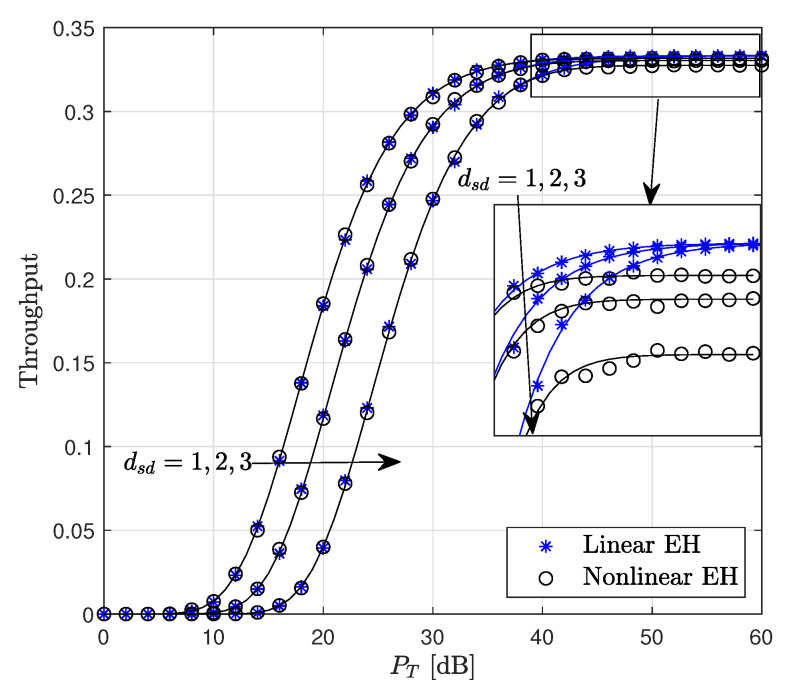
Throughput performance of the WP DH AF relaying system versus $P_{T}$ (R=1 bits/s/Hz, $P_{th}=35$ dB).

Figure 8 depicts the throughput with respect to $P_{T}$ for different values of $d_{sd}$. The target rate is fixed at R=1. The results reveal that, for the distances $d_{sd}=1,2,3$, the linear EH model provides performance approximately equivalent to that of the non-linear EH since the amount of harvested powers are approximately equal for both EH models. Moreover, in all cases, the throughput reaches its maximum value at $P_{T}=42$ dB.

**Figure 9:**
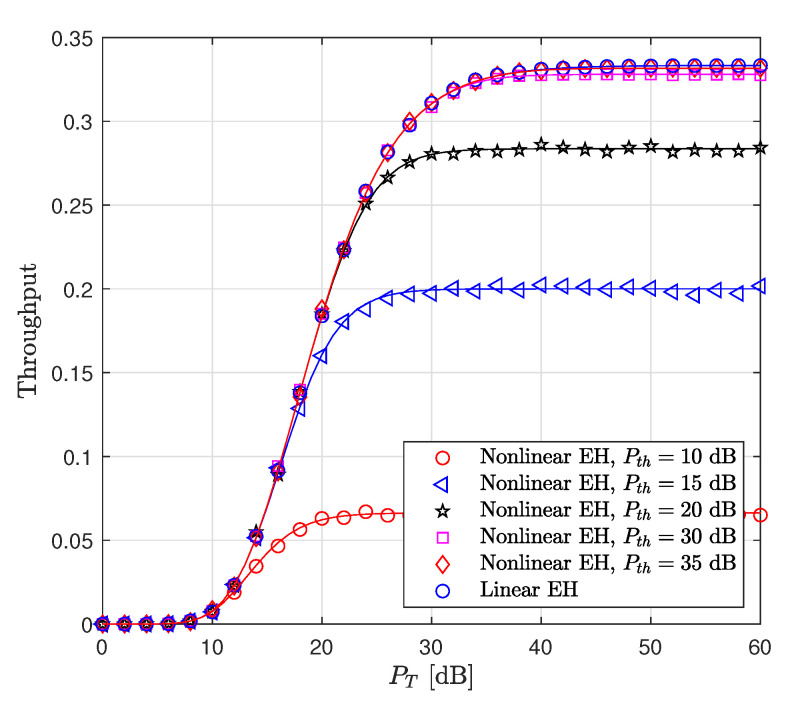
Throughput performance of the WP DH AF relaying system versus $P_{T}$ (R=1 bits/s/Hz, $d_{sd}=1$).

The throughput performance versus $P_{T}$ for different values of $P_{th}$ is shown in Figure 9. It is seen from Figure 9 that, by increasing $P_{th}$ from 10 dB to 35 dB, the throughput increases and reaches its maximum value for $P_{th}=35$ dB, which is approximately equivalent to the linear EH model throughput performance. In other words, a high level of threshold power in the non-linear EH model provides the same results as in the linear EH model since the amount of harvested power for both EH models is equal.

**Figure 10:**
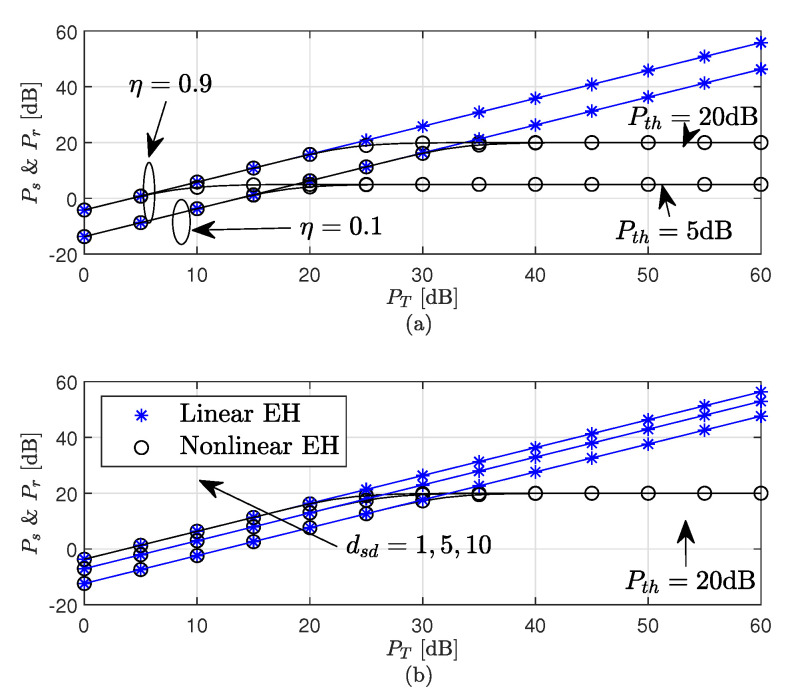
$P_{s}$ and $P_{r}$ of the WP DH AF relaying system (**a**) $d_{sd}=2$, (**b**) $\eta =\eta_{s}=\eta_{r}=0.9$.

Figure 10 plots the source and relay harvested powers versus $P_{T}$. Please note that, from Appendix D, for an equal distance of S and R from PB, and for equal fading channel parameters, the harvested powers $P_{s}$ and $P_{r}$ are equal. The curves in Figure 10 are obtained using (A34) and (A35) for linear and non-linear EH models, respectively. As seen from Figure 10a,b, the distance ($d_{sd}$) and energy efficiency (η) have a significant effect on the amount of powers harvested at S and R. It is observed that, for high values of $P_{T}$, the average harvested power in the linear EH model is higher compared to the considered non-linear EH model, which is saturated to a predefined threshold value $P_{th}$. This high value is contrary to the amount of harvested energy in practical NL EH models, where it results in an overestimation of system performance.

**Figure 11:**
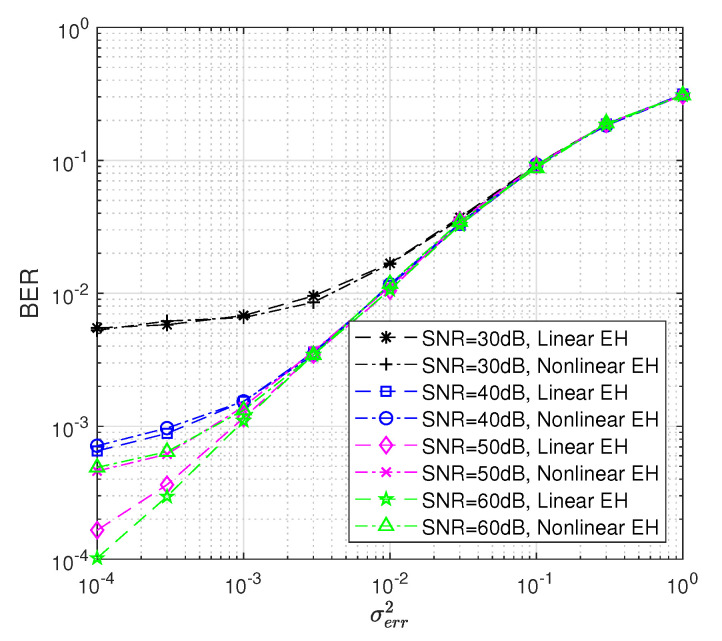
BER performance of the WP DH AF relaying system versus $P_{T}$ dB where $P_{th}$ = 35 dB, $d_{sd}=1$.

Figure 11 shows the BER performance versus channel estimation error power $\sigma_{err}^{2}$ for different SNR values. Both linear and non-linear EH results are obtained considering $P_{th}=35$ dB and $d_{sd}=1$. Moreover, we modeled the imperfect CSI case as $\hat{g_{1}}=g_{1}+e$ and $\hat{g_{2}}=g_{2}+e$, where *e* denotes the the channel estimation error with distribution $\mathrm{CN}∼(0,\sigma_{err}^{2})$, $\sigma_{err}^{2}$ denoting the power of the channel estimation error. It is shown that system performances get worse when the power of channel estimation error increases for both linear and non-linear EH models.

## 7. Conclusions

In this paper, a WP DH AF relaying system has been considered and a comprehensive performance analysis has been undertaken considering linear and non-linear EH models. In the studied system, S and R have been assumed to be battery-less, and that they harvest their power from a dedicated PB. For a comprehensive analysis, the results have been obtained considering the non-linear EH model together with the linear EH model which is mostly assumed in the literature. The BEP and throughput of the system for both linear and non-linear EH models have been analytically derived and compared with Monte Carlo simulation results, which verify our theoretical derivations. Moreover, results have been provided for different system parameters. It has been shown that the linear EH model misrepresents the system performance at high amounts of harvested energy and only provides reasonable results at low amounts of harvested energy. Hence, this causes an inaccurate understanding of the process of EH system design. However, the studied non-linear EH model provides a realistic result for the system performance, both at low and high harvested energy levels, which provides a comprehensive insight into the EH system architecture. We considered the case of a single antenna in all nodes in this paper. However, it is possible to have more antennas at the S, R, and D nodes and to analyze the improved system performance based on transmit antenna selection and maximum ratio combining. The analysis of these systems will be part of future work.

## Figures and Tables

**Table 1:** List of notations.

Notation	Description
Γ(·,·)	Upper incomplete Gamma function ([50], 8.350)
$F_{\cdot }\left(\cdot \right)$	Cumulative distribution function (CDF)
$f_{\cdot }\left(\cdot \right)$	Probability distribution function (PDF)
Γ(·)	Gamma function ([50], 8.310).
γ(·,·)	Lower incomplete Gamma function ([50], 8.350)
${\left(\cdot \right)}_{\cdot }$	Pochhammer’s symbol ([51], 6.1-22)
${}_{1}F_{1}\left(\cdot ;\cdot ;\cdot \right)$	Kummer Confluent Hyper-geometric function ([52], 07.20.07.0001.01)
$G\begin{array}{c} mn\\ pq \end{array}\left(\cdot |\begin{array}{c} a_{p}\\ b_{q} \end{array}\right)$	Meijer-G function ([50], 9.301)
$K_{x}\left(\cdot \right)$	Modified Bessel function of the second kind with order *x* [50]

**Table 2:** List of parameters.

Parameters	Description
$P_{T}$	PB transmit power
$\eta_{s}$	Energy conversion coefficient $\left(0<\eta_{s}<1\right)$
$\eta_{r}$	Energy conversion coefficient $\left(0<\eta_{r}<1\right)$
$P_{s}$	Harvested power at node S
$P_{r}$	Harvested power at node R
*x*	Transmitted signal from S with $E\{|x{|}^{2}\}=1$
a,b	Modulation specific constants [4]
$\Omega_{h_{i}}=E\{|h_{i}{|}^{2}\}$	Channel fading gains, i∈{s,r}
$\Omega_{g_{j}}=E\{|g_{j}{|}^{2}\}$	Channel fading gains, j∈{1,2}
$P_{th_{i}}$	Threshold (saturation) power at source and relay nodes i∈{s,r}
ν	Path loss exponent
$L_{i}=1/\sqrt{1+d_{i}^{\nu }}$	Pathloss i∈{st,rt,sr,rd} [1,4]
$d_{sr}$	S→R link distance
$d_{rd}$	R→D link distance
$d_{st}$	Distances from PB to S
$d_{rt}$	Distances from PB to R
$d_{v}$	Vertical distance from PB to node S
$d_{h}$	Horizontal distance from S to PB
$N_{0}$	Noise power spectral density
$\vartheta =\log_{2}M$	Number of bits per symbol
*M*	Modulation order

## Data Availability

The data leading to the results presented in this study are available on request from the corresponding author.

## Abbreviations

The following abbreviations are used in this manuscript:

AF	Amplify-and-forward
AWGN	Additive white Gaussian noise
BEP	Bit error probability
BER	Bit error rate
CDF	Cumulative distribution function
CR	Cognitive radio
CSI	Channel state information
D	Destination
DF	Decode-and-forward
DH	Dual-hop
EH	Energy harvesting
ER	Energy receiver
FD	Full-duplex
IoT	Internet of Things
i.i.d	Independent and identically distributed
LoS	Line of sight
MIMO	Multiple-input multiple-output
NOMA	Non-orthogonal multiple access
OFDM	Orthogonal frequency-division multiplexing
PB	Power beacon
PDF	Probability distribution function
PS	Power-splitting
PT	Primary transmitter
R	Relay
RF	Radio frequency
r.v	Random variables
S	Source
SEP	Symbol error probability
ST	Secondary transmitter
SWIPT	Simultaneous wireless information and power transfer
TS	Time-switching
TW	Two-way
UAV	Unmanned aerial vehicle
WP	Wireless powered
WPC	Wireless-powered communication.

## Appendix A. Calculation of *A*(*w*)

In (16), substituting (14), A(w) is re-expressed as

(A1) $$\begin{array}{c} A\left(w\right)=\frac{a}{2}\int_{0}^{P_{th}}\left[1-\frac{1}{\sqrt{C+\frac{B}{Z}}}\right]f_{Z}\left(z\right)dz, \end{array}$$

where $C=\left(1+w\bar{y}\right)/w\bar{y}$, $B=1/\bar{x}$ and $P_{th}=P_{th_{s}}=P_{th_{r}}$ and $f_{Z}\left(z\right)$ is given in (21). Substituting (21), (A1) is written as

(A2) $$\begin{array}{c} A\left(w\right)=\frac{a}{2}\left(E_{1}-F_{1}\left(w\right)\right), \end{array}$$

where

(A3) $$\begin{array}{c} E_{1}=\int_{0}^{P_{th}}f_{Z}\left(z\right)dz=D\gamma \left(m_{z},P_{th}\phi_{z}\right)/\phi_{z}^{m_{z}} \end{array}$$

and after simplifying,

(A4) $$\begin{array}{c} F_{1}\left(w\right)=\frac{D}{\sqrt{B}}\int_{0}^{P_{th}}\frac{z^{m_{z}-0.5}}{\sqrt{1+z/G}}\exp\left(-z\phi_{z}\right)dz, \end{array}$$

where G=B/C. Moreover, (A3) is calculated using ([50], 3.351-1). Taking z/G=x and after some mathematical modifications, (A4) is given as

(A5) $$\begin{array}{c} F_{1}\left(w\right)=\frac{D}{\sqrt{B}}\left[M(w)+N(w)\right]. \end{array}$$

Defining

(A6) $$\begin{array}{c} T_{i}\left(w\right)=G^{m_{z}+0.5}\int_{x_{min}^{i}}^{x_{max}^{i}}\frac{x^{m_{z}-0.5}}{\sqrt{1+x}}\exp\left(-\phi_{z}Gx\right)dx \end{array}$$

and taking the integral limits as $\left\{x_{min}^{1}=0,x_{max}^{1}=1\right\}$ and $\left\{x_{min}^{2}=1,x_{max}^{2}=P_{th}/G\right\}$ for i=1 and i=2, respectively, we have $M\left(w\right)=T_{1}\left(w\right)$ and $N\left(w\right)=T_{2}\left(w\right)$. Using ([56], eq.3-a) and ([52], 07.20.07.0001.01) for i=1 and using ([56], eq.3-b) and ([50], eq. 3.351-2) for i=2 in (A6), M(w) and N(w) in (A5) are calculated as

(A7) $$\begin{array}{cc} M(w) & =G^{m_{z}+0.5}\sum_{k=0}^{\Upsilon }{\left(0.5\right)}_{k}\frac{{\left(-1\right)}^{k}}{k!}\int_{0}^{1}x^{m_{z}+k-0.5}e^{-\phi_{z}Gx}dx\\ \; & =G^{m_{z}+0.5}\sum_{k=0}^{\Upsilon }{\left(0.5\right)}_{k}\frac{{\left(-1\right)}^{k}}{k!}\frac{\Gamma \left(1\right)\Gamma \left(m_{z}+k+0.5\right)}{\Gamma (m_{z}+k+1.5)}\\ \; & \times {}_{1}F_{1}\left(m_{z}+k+0.5;m_{z}+k+1.5;-\phi_{z}G\right) \end{array}$$

and

(A8) $$\begin{array}{cc} N(w) & =G^{m_{z}+0.5}\sum_{j=0}^{\Upsilon }{\left(0.5\right)}_{j}\frac{{\left(-1\right)}^{j}}{j!}\int_{1}^{P_{th}/G}x^{m_{z}-1-j}\exp\left(-\phi_{z}Gx\right)dx\\ \; & =\sum_{j=0}^{\Upsilon }{\left(0.5\right)}_{j}\frac{{\left(-1\right)}^{j}}{j!}\frac{\Gamma \left(m_{z}-j,\phi_{z}G\right)-\Gamma \left(m_{z}-j,\phi_{z}P_{th}\right)}{G^{-(j+0.5)}\phi_{z}^{\left(m_{z}-j\right)}}. \end{array}$$

Finally, (A5) is calculated by substituting (A7) and (A8). Then, (A2) is calculated by replacing (A3) and (A5). As seen from (A2), $E_{1}$ is independent of *w* whereas $F_{1}$ is a function of *w*.

## Appendix B. Calculation of *P*_*s*1_

Considering ([51], eq. 13.1.12), ([50], eq. 8.338-1), ([50], eq. 8.331-1) and ([51], eq. 6.1-22), (A7) is simplified. Moreover, G=B/C, $B=1/\bar{x}$ and $C=\left(1+w\bar{y}\right)/w\bar{y}$ are replaced in (A7) and (A8). After simplification, (A5) is given as

(A9) $$\begin{array}{c} F_{1}\left(w\right)=\frac{D}{\sqrt{B}}\left[M\left(w\right)+N_{1}\left(w\right)-N_{2}\left(w\right)\right], \end{array}$$

where

(A10) $$\begin{array}{cc} M(w) & =\Lambda \left(k,n\right){\left(\frac{w\bar{y}}{1+w\bar{y}}\right)}^{n+m_{z}+0.5}, \end{array}$$

(A11) $$\begin{array}{c} N_{1}\left(w\right)=\sum_{j=0}^{\Upsilon }{\left(0.5\right)}_{j}\frac{{\left(-1\right)}^{j}}{j!}\phi_{z}^{-(m_{z}-j)}{\left(\frac{Bw\bar{y}}{1+w\bar{y}}\right)}^{j+0.5}\Gamma \left(m_{z}-j,\phi_{z}\frac{Bw\bar{y}}{1+w\bar{y}}\right) \end{array}$$

and

(A12) $$\begin{array}{c} N_{2}\left(w\right)=\beta \left(j\right){\left(\frac{w\bar{y}}{1+w\bar{y}}\right)}^{j+0.5}. \end{array}$$

In (A10) and (A12), we have

(A13) $$\begin{array}{c} \Lambda \left(k,n\right)=\sum_{k=0}^{\Upsilon }\sum_{n=0}^{\Upsilon }\frac{{\left(0.5\right)}_{k}{\left(-1\right)}^{k}{\left(-1\right)}^{n}\phi_{z}^{n}}{k!n!(m_{z}+k+n+0.5)}B^{n+m_{z}+0.5} \end{array}$$

and

(A14) $$\begin{array}{c} \beta \left(j\right)=\sum_{j=0}^{\Upsilon }{\left(0.5\right)}_{j}\frac{{\left(-1\right)}^{j}B^{j+0.5}\Gamma \left(m_{z}-j,\phi_{z}P_{th}\right)}{j!\phi_{z}^{\left(m_{z}-j\right)}}, \end{array}$$

where *k*, *n* and *j* are the indices of infinite summations defined in Λ(k,n) and β(j), respectively. Using ([52], 06.06.26.0005.01) and ([50], eq. 9.931-5), simplifying and substituting in (A11), we have

(A15) $$\begin{array}{c} N_{1}\left(w\right)=\alpha \left(j\right)G\begin{array}{c} 20\\ 12 \end{array}\left(\phi_{z}B\frac{w\bar{y}}{1+w\bar{y}}|\begin{array}{c} j+1.5\\ j+0.5,m_{z}+0.5 \end{array}\right), \end{array}$$

where

(A16) $$\begin{array}{c} \alpha \left(j\right)={\left(\frac{1}{\phi_{z}}\right)}^{m_{z}+0.5}\sum_{j=0}^{\Upsilon }{\left(0.5\right)}_{j}\frac{{\left(-1\right)}^{j}}{j!}. \end{array}$$

Finally, the simplified version of $F_{1}\left(w\right)$ in (A9) is calculated by replacing (A10), (A15) and (A12). Then, substituting (A9) and (A3) in (A2), and, after some mathematical simplifications, $P_{s1}$ in (16) is given as

(A17) $$\begin{array}{c} P_{s1}=\int_{0}^{P_{th}}A\left(w\right)f_{W}\left(w\right)dw=\frac{a}{2}\left[E_{1}E_{2}-L\right], \end{array}$$

where

(A18) $$\begin{array}{c} E_{1}E_{2}=DI\gamma \left(m_{z},P_{th}\phi_{z}\right)\gamma \left(m_{w},P_{th}\phi_{w}\right)/\phi_{w}^{m_{w}}\phi_{z}^{m_{z}} \end{array}$$

and

(A19) $$\begin{array}{c} L=\frac{D}{\sqrt{B}}\left[L_{1}+L_{2}-L_{3}\right]. \end{array}$$

(A18) is calculated by substituting (A3) and (A30). In (A19), substituting (A10), (A15) and (A12) and, after mathematical simplifications, we have

(A20) $$\begin{array}{cc} L_{1} & =\int_{0}^{P_{th}}M\left(w\right)f_{W}\left(w\right)dw=I\Lambda \left(k,n\right)\Delta_{1}, \end{array}$$

(A21) $$\begin{array}{cc} L_{2} & =\int_{0}^{P_{th}}N_{1}\left(w\right)f_{W}\left(w\right)dw=I\alpha \left(j\right)\int_{0}^{P_{th}}w^{m_{w}-1}\exp\left(-w\phi_{w}\right)\\ & \times G\begin{array}{c} 20\\ 12 \end{array}\left(\phi_{z}B\frac{w\bar{y}}{1+w\bar{y}}|\begin{array}{c} j+1.5\\ j+0.5,m_{z}+0.5 \end{array}\right)dw \end{array}$$

and

(A22) $$\begin{array}{cc} L_{3} & =\int_{0}^{P_{th}}N_{2}\left(w\right)f_{W}\left(w\right)dw=I\beta \left(j\right)\Delta_{2}. \end{array}$$

Please note that (A21) is calculated numerically since a closed-form solution is not tractable. In (A20) and (A22), we have

(A23) $$\begin{array}{c} \Delta_{i}=\int_{0}^{P_{th}}w^{m_{w}-1}\exp\left(-w\phi_{w}\right){\left(\frac{w\bar{y}}{1+w\bar{y}}\right)}^{u_{i}}dw, \end{array}$$

where for i={1,2}, we define $u_{1}=n+m_{z}+0.5$ and $u_{2}=j+0.5$, respectively, for (A20) and (A22). Taking $x=w\bar{y}$ and simplifying (A23), (A20) and (A22) are rewritten as

(A24) $$\begin{array}{cc} L_{1} & =I\Lambda \left(k,n\right){\left(\frac{1}{\bar{y}}\right)}^{m_{w}}\left(\lambda_{1}+\varpi_{1}\right) \end{array}$$

and

(A25) $$\begin{array}{cc} L_{3} & =I\beta \left(j\right){\left(\frac{1}{\bar{y}}\right)}^{m_{w}}\left(\lambda_{2}+\varpi_{2}\right), \end{array}$$

respectively, where

(A26) $$\begin{array}{cc} \lambda_{v} & =\int_{0}^{1}x^{m_{w}+u_{v}-1}{\left(1+x\right)}^{-u_{v}}\exp\left(-x\phi_{w}/\bar{y}\right)dx\\ \; & =\sum_{\delta =0}^{\Upsilon }{\left(u_{v}\right)}_{\delta }\frac{{\left(-1\right)}^{\delta }}{\delta !}\int_{0}^{1}x^{m_{w}+u_{v}+\delta -1}\exp\left(-x\phi_{w}/\bar{y}\right)dx\\ \; & =\sum_{\delta =0}^{\Upsilon }{\left(u_{v}\right)}_{\delta }\frac{{\left(-1\right)}^{\delta }}{\delta !}\frac{1}{m_{w}+u_{v}+\delta }{}_{1}F_{1}\left(m_{w}+u_{v}+\delta ;m_{w}+u_{v}+\delta +1;-\frac{\phi_{w}}{\bar{y}}\right). \end{array}$$

(A26) is calculated and simplified using ([56], eq. 3-a), ([51], eq. 13.2.1) and considering ([50], eq. 8.338-1) and ([50], eq. 8.331-1). Please note that in (A26), v=1 and v=2 for (A24) and (A25), respectively. Moreover, in (A24) and (A25),

(A27) $$\begin{array}{cc} \varpi_{v} & =\int_{1}^{P_{th}\bar{y}}x^{m_{w}+u_{v}-1}{\left(1+x\right)}^{-u_{v}}\exp\left(-x\phi_{w}/\bar{y}\right)dx\\ \; & =\sum_{\xi =0}^{\Upsilon }{\left(u_{v}\right)}_{\xi }\frac{{\left(-1\right)}^{\xi }}{\xi !}{\left(\frac{\bar{y}}{\phi_{w}}\right)}^{m_{w}-\xi }\left(\Gamma \left(m_{w}-\xi ,\frac{\phi_{w}}{\bar{y}}\right)-\Gamma \left(m_{w}-\xi ,\phi_{w}P_{th}\right)\right). \end{array}$$

(A27) is calculated using ([56], eq. 3-b) and ([50], eq. 3.351-2). (A24) and (A25) are calculated by substituting (A26) and (A27), respectively, for v=1 and v=2. (A19) is calculated by substituting (A24), (A21) and (A25). Finally, (A17) is calculated by substituting (A19) and (A18). As seen from (A17), $P_{s1}$ is dependent on *L*, $E_{1}$ and $E_{2}$.

## Appendix C. Calculation of *A*(*z*)

In (19), substituting (14), A(z) is written as

(A28) $$\begin{array}{c} A\left(z\right)=\frac{a}{2}\int_{0}^{P_{th}}\left[1-\frac{1}{\sqrt{C^{\prime }+\frac{B^{\prime }}{W}}}\right]f_{W}\left(w\right)dw, \end{array}$$

where $C^{\prime }=\left(1+z\bar{y}\right)/z\bar{y}$, $B^{\prime }=1/\bar{y}$, and $f_{W}\left(w\right)$ is given in (23). Noting that A(z) given in (A28) is a function with parameter *z* and applying the same procedure from (A2) to (A8), we have

(A29) $$\begin{array}{c} A\left(z\right)=\frac{a}{2}\left(E_{2}-F_{2}\left(z\right)\right), \end{array}$$

where

(A30) $$\begin{array}{c} E_{2}=\int_{0}^{P_{th}}f_{W}\left(w\right)dw=I\gamma \left(m_{w},P_{th}\phi_{w}\right)/\phi_{w}^{m_{w}} \end{array}$$

and

(A31) $$\begin{array}{c} F_{2}\left(z\right)=\frac{I}{\sqrt{B^{\prime }}}\left[M^{\prime }\left(z\right)+N^{\prime }\left(z\right)\right]. \end{array}$$

In (A31),

(A32) $$\begin{array}{cc} M^{\prime }\left(z\right) & =G_{2}^{m_{w}+0.5}\sum_{k=0}^{\Upsilon }{\left(0.5\right)}_{k}\frac{{\left(-1\right)}^{k}}{k!}\frac{\Gamma \left(1\right)\Gamma \left(m_{w}+k+0.5\right)}{\Gamma (m_{w}+k+1.5)}\\ & \times {}_{1}F_{1}\left(m_{w}+k+0.5;m_{w}+k+1.5;-\phi_{w}G_{2}\right) \end{array}$$

and

(A33) $$\begin{array}{c} N^{\prime }\left(z\right)=\sum_{j=0}^{\Upsilon }{\left(0.5\right)}_{j}\frac{{\left(-1\right)}^{j}\left(\Gamma \left(m_{w}-j,\phi_{w}G_{2}\right)-\Gamma \left(m_{w}-j,\phi_{w}P_{th}\right)\right)}{j!G_{2}^{-(j+0.5)}\phi_{w}^{\left(m_{w}-j\right)}}, \end{array}$$

where $G_{2}=B^{\prime }/C^{\prime }$. Please note that $E_{2}$ is independent of *z* in (A29).

## Appendix D. Calculation of Average Powers

The average harvested powers at S and R are given as

(A34) $$\begin{array}{c} P^{L}\left(\Xi ,i,\rho \right)=\int_{0}^{\infty }\rho f_{\rho }\left(\rho \right)d\rho =\Xi \Gamma \left[m_{\rho }+1\right]/\phi_{\rho }^{m_{\rho }+1} \end{array}$$

and

(A35) $$\begin{array}{c} P^{NL}\left(\Xi ,i,\rho \right)=\int_{0}^{P_{th}}\rho f_{\rho }\left(\rho \right)d\rho +P_{th}B_{i}=\Xi \gamma \left(m_{\rho }+1,P_{th}\phi_{\rho }\right)/\phi_{\rho }^{m_{\rho }+1}+P_{th}B_{i} \end{array}$$

for linear and non-linear EH models, and are calculated by applying ([50], 3.326-2) and ([50], 3.351-1), respectively. Considering (A34), the average powers at both S and R for the linear EH model are $P_{s}^{L}=P^{L}\left(D,2,z\right)$ and $P_{r}^{L}=P^{L}\left(I,1,w\right)$, while the average powers of S and R for the non-linear EH model are obtained considering (A35) as $P_{s}^{NL}=P^{L}\left(D,2,z\right)$ and $P_{r}^{NL}=P^{L}\left(I,1,w\right)$, respectively. Here, $f_{Z}\left(z\right)$ and $f_{W}\left(w\right)$ are given in (21) and (23) and $B_{1}$ as well as $B_{2}$ are defined and calculated in (22) and (20), respectively.
